# Synthesis of Silver Embedded Poly(o-Anisidine) Molybdophosphate Nano Hybrid Cation-Exchanger Applicable for Membrane Electrode

**DOI:** 10.1371/journal.pone.0096897

**Published:** 2014-05-07

**Authors:** Anish Khan, Aftab Aslam Parwaz Khan, Abdullah M. Asiri, Malik Abdul Rub

**Affiliations:** 1 Chemistry Department, King Abdulaziz University, Jeddah, Saudi Arabia; 2 Center of Excellence for Advanced Materials Research (CEAMR), King Abdulaziz University, Jeddah, Saudi Arabia; Martin-Luther-Universität Halle-Wittenberg, Germany

## Abstract

Poly(o-anisidine) molybdophosphate was expediently obtained by sol-gel mixing of Poly(o-anisidine) into the inorganic matrices of molybdophosphate, which was allowed to react with silver nitrate to the formation of poly(o-anisidine) molybdophosphate embedded silver nano composite. The composite was characterized by Fourier Transform Infrared Spectroscopy, X-ray powder diffraction, UV-Vis Spectrophotometry, Fluorescence Spectroscopy, Scanning Electron Microscopy/Energy-dispersive X-ray Spectroscopy and Thermogravimertic Analysis. Ion exchange capacity and distribution studies were carried out to understand the ion-exchange capabilities of the nano composite. On the basis of highest distribution studies, this nano composite cation exchanger was used as preparation of heavy metal ion selective membrane. Membrane was characterized for its performance as porosity and swelling later on was used for the preparation of membrane electrode for Hg(II), having better linear range, wide working pH range (2–4.5) with fast response in the real environment.

## Introduction

Organic-inorganic hybrid materials, based on interactions between organic and inorganic components, have been extensively developed in the past decades [1]–[4]. The inorganic components include zeolite [5], layered structures [6]–[7], sometimes one dimensional polymers [8]; and the organic component could be small organic molecules, organometallics or organic polymers. The resultant hybrids may exhibit properties synergistically derived from the two components [9]. Molybdophosphate has interesting properties and practical applications, such as catalysts and cathode materials. Van der Waals bonds connect the nearest layers. Inserting guests (Ag) into the host will modify the properties of MoP and have interesting applications. Due to their anisotropic optical and electrical properties, electrochemical and electrochromic behaviors, conjugated polymers such as polypyrrole, polyaniline and polythiophene [10] have been used.

Generally, the synthetic methodology of a nanocomposite depends on the chemical and physical properties of the host inorganic materials and the guest organic polymers [11], [12]. It has been believed that the guest species needs to be soluble in some solvent system, whether miscible or immiscible with water. To date, most of polymer nanocomposites have been achieved through the direct insertion approach, where the polymers are first dispersed in water or organic solvent and then inserted into the layered structure. Another synthetic approach involves insertion of monomer first, followed by treatment with an oxidant. In this paper, this approach has been developed to prepare the intercalation of poly(ortho-methoxy aniline) POMA into MoP.

Design, fabrication and application of novel electrochemical sensors have been a topic of research in recent years [13], [14]. For this aspect, modification of membranes with suitable functionalities is an ongoing task among researchers world-wide because of its ability to improve the ion transfer rate from substrates to the membrane [15], [16]. In this topic of research, modification of membrane with heteropolyacids (HPAs) has received much attention [17], [18] owing to their attractive electronic and molecular properties, which results in novel applications in catalysis, materials science [19] and energy storage devices [20], [21] etc. However, the high solubility of heteropolyacids in aqueous media limits the stability of those modified membranes, as it leads to leaching of hetero polycation from the membrane surface and to the consequent drop of their electrochemical features. Composite prepared of inorganic matrix and conducting polymer have reduced leaching of exchanger due to its interactions with the polymer matrix and the poor solubility of the conducting polymer in water. In addition, inorganic clusters keep their integrity and activity while benefiting from the conducting properties and polymeric nature of the hybrid structure. Further incorporation of metal nanoparticles into the organic-inorganic hybrid materials offers enhanced performance due to the increase in conductivity and surface area. In this regard, we made an attempt to fabricate membranes with metal-incorporated organic-inorganic hybrid material for ion selective electrode, i.e. silver-incorporated POMA-MoP. Hence in the present paper, we developed a facile method to fabricate a metal-incorporated organic–inorganic composite-embedded membrane and its electrochemical properties.

## Experimental

### Reagents

The main reagents used for the synthesis of the material were obtained from CDH, Loba Chemie, E-merck and Qualigens (used as received). All other reagents and chemicals were of analytical grade.

### Preparation of POMA-MoP/Ag

The polymerization of the monomer ortho-anisidine was initiated by the addition of oxidizing agent, i.e., ammonium persulfate in 1∶1 ratio under constant stirring at 5°C for 6 h. After 6 h a greenish color poly-o-anisidine polymer obtained [22]. The method of preparation of the inorganic precipitate of MoP ion-exchanger was very similar to that of Alberty and co-author [23] and Constantino [24] with slight modification.

The composite was prepared by the sol-gel mixing of POMA, an organic polymer into the inorganic matrices of MoP. In this process when the gels of POMA were added to the inorganic MoP with a constant stirring, the resultant mixture was turned slowly into brown colored slurries and kept for 24 hours at room temperature.

Now the resultant composite gels were filtered off, washed thoroughly with demineralized water (DMW) to remove excess acid and any adhering trace of oxidant. The washed material was dried over P_4_O_10_ at 45°C in an oven. The dried products were immersed in DMW to obtain small granules. They were converted to the H^+^ form by keeping them into 1 M HNO_3_ solution for 24 hours with occasionally shaking intermittently replacing the supernatant liquid. The excess acid was removed after several washing with DMW. The material was finally dried at 45°C and sieving to obtain particles of particular size range (∼125 µm). Hence a number of POMA-MoP/Ag nanocomposite cation-exchanger samples were prepared and on the basis of Na^+^ ion-exchange capacity (i.e.c.), and physical appearance, best one sample was selected for further studies.

### Ion-exchange Capacity (i.e.c.)

The ion-exchange capacity, which is generally taken as a measure of the hydrogen ion liberated by neutral salt to flow through the composite cation-exchanger was determined by standard column process.

### Thermal Studies

Simultaneous thermogravimetric analysis (TGA) studies of the composite cation-exchanger (POMA-MoP/Ag) in original form were carried out by an automatic thermo balance on heating the material from 10°C to 600°C at a constant rate (10°C per min) in the air atmosphere (air flow rate of 400 mL min^−1^).

### Characterization

The FT-IR (Spectrum-100 FT-IR) spectrum of POMA-MoP/Ag in original form, dried at 40°C, was taken by KBr disc method at room temperature. UV-visible studies were carried out by Lamda-950, Perkin Elmer, Germany. Powder X-ray diffraction (XRD) pattern was obtained in an aluminum sample holder in original form using a PW 1148/89 based diffractometer with Cu Kα radiations. Microphotographs of the original form of POMA-MoP/Ag were obtained by the FE-SEM (FESEM; JSM-7600F, Japan) at various magnifications.

### Selectivity (Sorption) Studies

The distribution coefficient (*K_d_* values) of various metal ions on POMA-MoP/Ag nano-composite were determined by batch method in various solvent systems [25]. The distribution coefficient (*K_d_*) was determined by using the following equation:
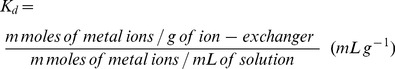
(1)




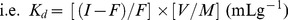
(2)where, *I* is the initial amount of metal ion in the aqueous phase, *F* is the final amount of metal ion in the aqueous phase, *V* is the volume of the solution (mL) and *M* is the amount of cation-exchanger (*g*).

### Preparation of POMA-MoP/Ag Cation-exchange Membrane

Ion-exchange membrane of POMA-MoP/Ag was prepared as the method reported by A.A. Khan and A. Khan [26] in earlier studies. To find out the optimum membrane composition, different amount of the composite (M-1 = 0.2 g, M-2 = 0.4 g, M-3 = 0.6 g) material was grounded to a fine powder and mixed thoroughly with a fixed amount of PVC (25%) dissolved in 10 mL tetrahydrofuran with one drop of plasticizers (Dioctyl Phthalate (DAP)). The resultant slurries were poured to cast in glass tube having 10 cm in length 5 mm in diameter. These glass tubes were left for slow evaporation for several hours. In this way, three sheets of different thicknesses 0.37, 0.39 and 0.40 mm were obtained. A fixed area of the membranes was cutted using sharp edge blade.

### Characterization of Membrane

For the characterization of membrane, three important parameters such as water content, porosity and thickness of membrane are determined as previously described in the literatures [27], [28].

#### Water content (% total wet weight)

First, the membranes were soaked in water to elute diffusible salt, blotted quickly with Whatmann filter paper to remove surface moisture and immediately weighted. These were further dried to a constant weight in a vacuum over P_2_O_5_ for 24 h. The water content (total wet weight) was calculated as:
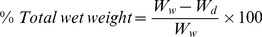
(3)where *W_d_* = weight of the dry membrane and *W_w_* = weight of the soaked/wet membrane.

#### Porosity

Porosity (*ε*) was determined as the volume of water incorporated in the cavities per unit membrane volume from the water content data:
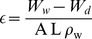
(4)where *W_w_* = weight of the soaked/wet membrane, *W_d_* = weight of the dry membrane, *A* = area of the membrane, *L* = thickness of the membrane and *ρ_w_* = density of water.

#### Thickness and swelling

The thickness of the membrane was measured by taking the average thickness of the membrane by using screw gauze. Swelling is measured as the difference between the average thicknesses of the membrane equilibrated with 1 M NaCl for 24h and the dry membrane.

### Fabrication of Ion-selective Membrane Electrode

The membrane sheet of 0.40 mm thickness (M-3) as obtained by the above procedure was cut in the shape of disc and mounted at the lower end of a pyrex glass tube (o.d. 0.8 cm, i.d. 0.6) with araldite. Finally, the assembly was allowed to dry in air for 24 h. The glass tube was filled with 0.1 M mercuric nitrate solution. A saturated calomel electrode was inserted in the tube for electrical contact and another saturated calomel electrode was used as external reference electrode. The whole arrangement can be shown as:
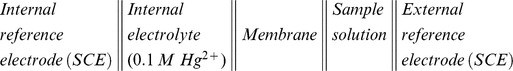



Following parameters were evaluated to study the characteristics of the membrane such as lower detection limit, response curve, response time and working pH range.

### Electrode Membrane Potential

To determine the membrane response, a series of standard solutions of varying concentrations ranging from 10^−1 ^M to 10^−10 ^M were prepared. External electrode and ion selective membrane electrode are plugged in digital potentiometer and the potentials were recorded.

For the determination of membrane potentials, the membrane electrode was conditioned by soaking in 0.1 M Hg(NO_3_)_2_ solution for 5–7 days and for 1 h before use. When membrane electrode was not in use it must be kept in 0.1 M Hg(NO_3_)_2_ solution. Potential measurement was plotted against selected concentration of the respective ion in aqueous solution.

### Effect of pH

pH solution ranging from 1–10 were prepared at constant ion concentration i.e (1×10^−2 ^M). The value of membrane potential at each pH was recorded and plot of membrane potential versus pH was plotted.

### The Response Time

The membrane is first dipped in a 1×10^−3 ^M solution of Hg(NO_3_)_2_ and then 10 fold higher concentration (1×10^−2 ^M). The potential of the solution was read at zero second; just after dipping of the membrane electrode in the second solution and subsequently recorded at the intervals of 30 s. The value of electrode potential at each pH was recorded and electrode potential was plotted vs. pH.

### Determination of Hg^2+^ by Potentiometric Titration using POMA-MoP/Ag Nano Composite Membrane Electrode

The practical utility of the proposed membrane electrode assembly was tested by its use as an indicator membrane electrode in the potentiometric titration of Hg(II) with ethylenediaminetetraaceticacid (EDTA).

## Results and Discussion

**Figure 1:**
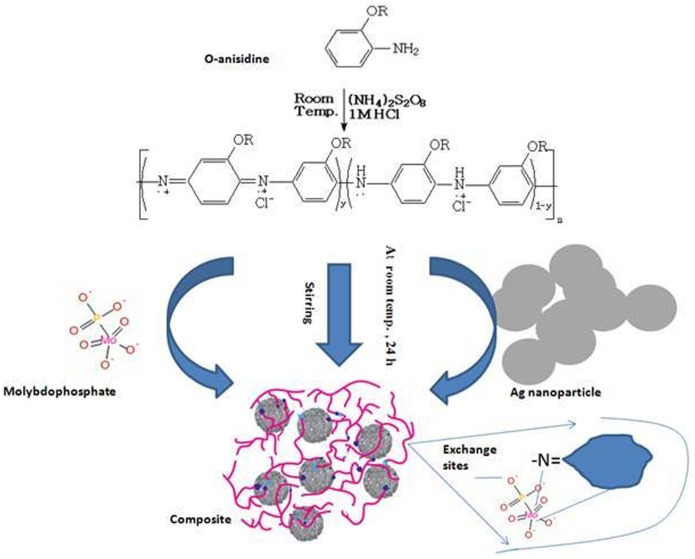
Schematic representation for the preparation of silver nano particle embeded poly(o-anisidine) molybdophosphate nano composite.

New and novel nano Ag embedded composite material has been synthesized by the incorporation of electrically conducting POMA into the inorganic matrices of MoP, responsible for cation exchange selectivity. Schematic representation for the preparation is given in **Fig. 1**. Due to the high percentage of yield, better ion-exchange capacity, reproducible behavior, chemical and thermal stabilities, best sample was chosen for detail studies. The composite cation-exchange material possessed a good Na^+^ ion-exchange capacity (1.25 meq g^−1^).

### Characterization

**Figure 2:**
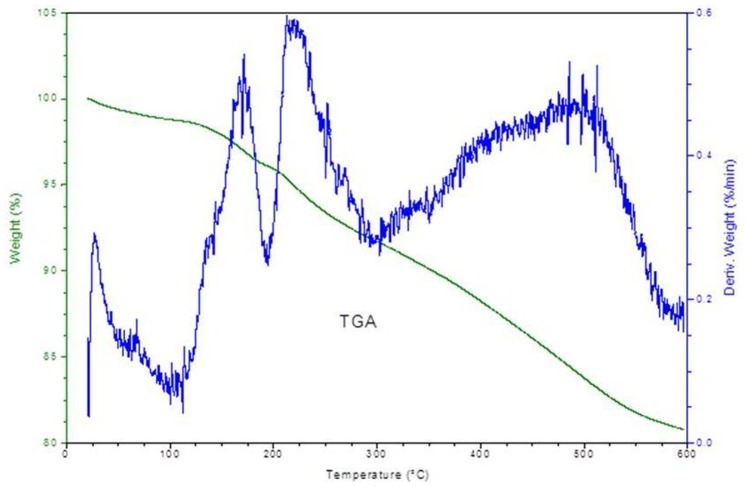
TGA curve of POMA-MoP/Ag nano composite cation exchanger.

It is clear from the TGA curve of the composite cation-exchanger that upto 125°C only 3% weight loss was observed, which may be due to the removal of external H_2_O molecules present at the surface of the composite [29]. Further continuous loss of mass approximately between 125 to 600°C may be due to the slight conversion of inorganic phosphate into pyrophosphate (**Fig. 2** **)** and may be due to the slight decomposition of organic part of the material.

**Figure 3:**
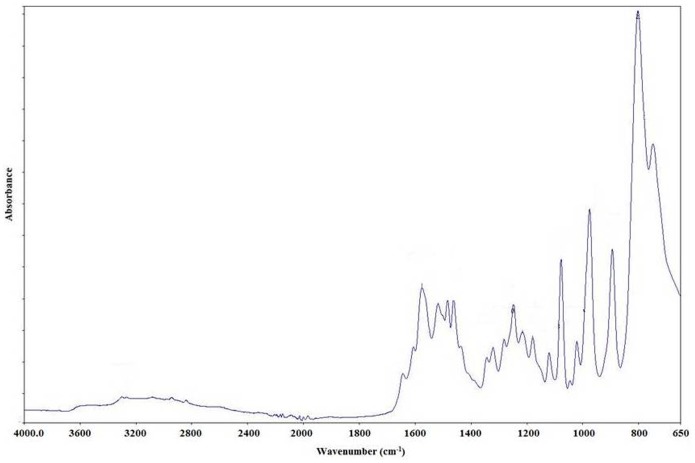
FT-IR spectra of pure POMA-MoP/Ag nano composite cation exchanger.

The FTIR spectrum of the composite cation-exchanger (**Fig. 3** **)** indicates the presence of external water molecule in addition to the –OH groups and metal oxides present internally in the material. The peak at the 1610 cm^−1^ may be due to interstitial water present in the composite material [30]. The peak at 1000 cm^−1^ may represent the presence of ionic phosphate groups [31] in the material. The additional band at between 1440 cm^−1^ can be ascribed to stretching vibration of C–N [32]. The characteristic peaks at around 1490 cm^−1^ and 1598 cm^−1^ are attributed to the stretching vibrations of N-B-N and N = Q = N rings respectively (where Q refers to Quinoid and B refers to Benzenoid ring).This indicates that the material contains considerable amount of OMA. The out of plane bending deformations of C-H in the substituted benzene ring are attributed to the band at 817 cm^−1^ that becomes stronger peaks due to the presence of metal effect.

**Figure 4:**
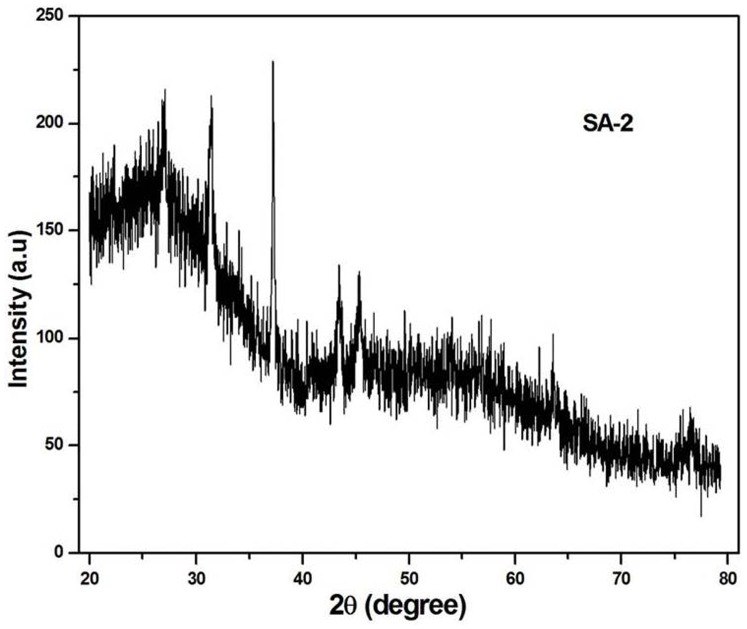
Powder X-ray diffraction spectra of POMA-MoP/Ag nanocomposite.

The phase identification for the prepared hybrid material was carried out using powder X-ray diffraction shown in **Fig. 4**. The observed XRD pattern of POMA-MoP/Ag hybrid semi crystalline and low resolved diffraction peaks (2θ) characteristic of silver. Diffraction peak at about 25° shows the characteristic formation of POMA which is clearly visible in the case of hybrid material whereas the observed intensity of the peak was very low in the case of POMA-MoP/Ag hybrid material. Reason for such low intensity is due to the presence of silver in POMA-MoP/Ag hybrid material which modified the relative ratio between the crystalline and amorphous structures. Further, no XRD diffraction patterns are noticed for bulk POMA-MoP/Ag phase indicates that the MoP is finely dispersed in the POMA matrix. The presence of nanosized silver particles in the hybrid material induces lattice strain which contributes broadening of XRD peaks. However, the crystallite size and strain contributions to line broadening are independent of each other [33]. Hence, the total peak broadening is represented by sum of the crystallite size and strain contributions and it is calculated from the X-ray diffraction pattern using Williamson–Hall equation as follows
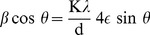
(5)


**Figure 5:**
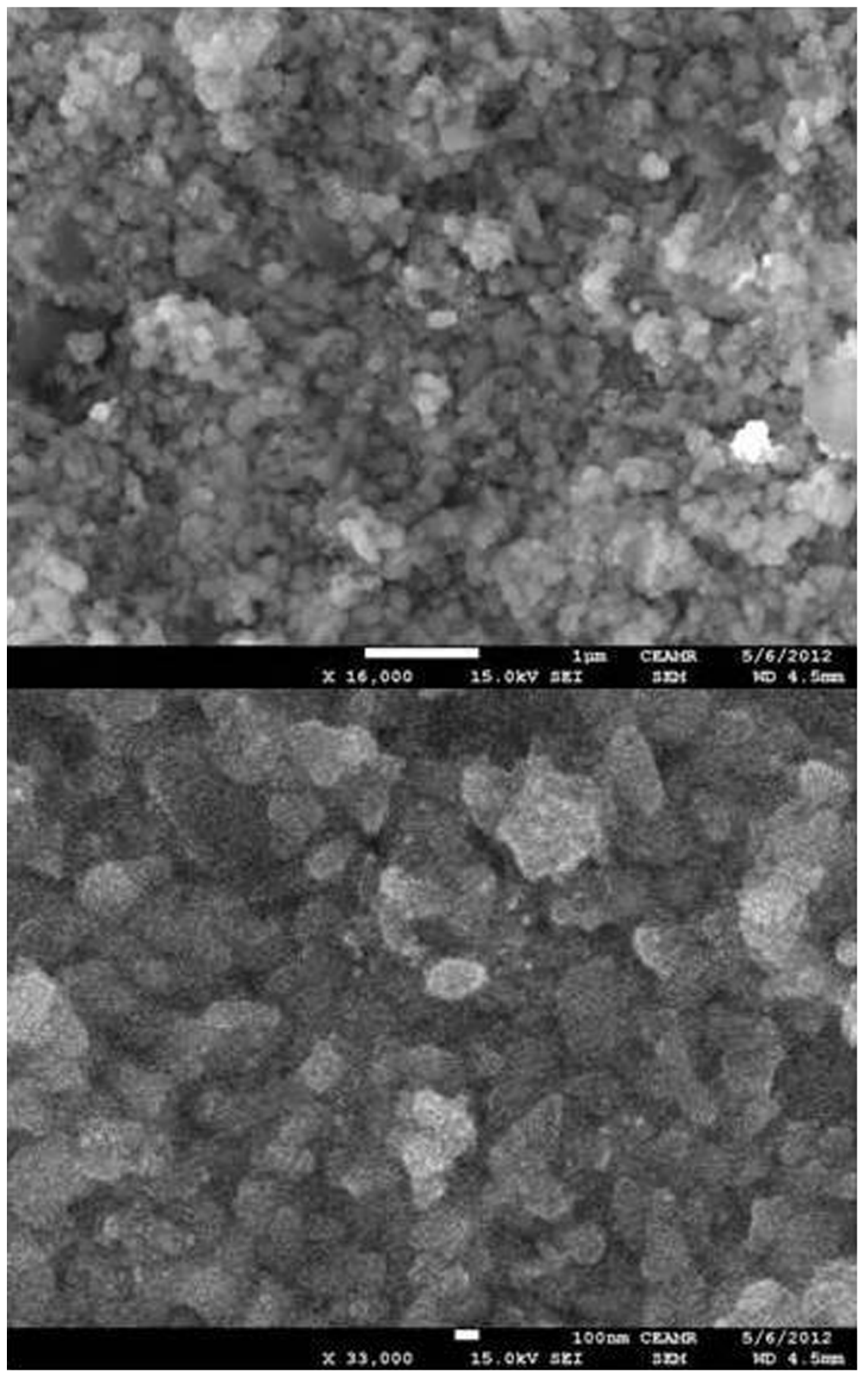
SEM images of pure POMA-MoP/Ag nano composite cation exchanger.

The SEM was employed to investigate the morphology and size of the nanoparticles in the hybrid materials. **Fig. 5a** shows the representative FE-SEM image of POMA-MoP/Ag. The formed silver nanoparticles are polydisperse and of irregular morphology with two size. **Fig. 5b** shows the high resolution FE-SEM image of one of the silver nanoparticles. The grey shadow present in the image may be due the MoP layer covered on the silver nanoparticles. Thus, the FE-SEM images provide further evidence for the presence of silver nanoparticles in the polymer hybrid matrix.

In order to explore the potential of the composite material in the separation of metal ions, distribution studies for different metal ions were performed in different solvent systems. It was observed that the *K_d_*- values vary with the nature of the contacting solvents. It was also observed from the (*K_d_*) values (**Table 1**) that Hg^2+^ was strongly adsorbed and Zn^2+^, Ba^2+^, Ca^2+^ and Al^3+^ are significantly adsorbed while the remaining are partially adsorbed on the surface of ion-exchange material.

**Table 1:** *K_d_*-values of some metal ions on POMA-MoP/Ag composite cation- exchanger column in different solvent systems.

Metals	Zn^2+^	Cu^2+^	Cd^2+^	Mn^2+^	Mg^2+^	Co^2+^	Ba^2+^	Sr^2+^	Pb^2+^	Hg^2+^
Solvents										
DMW	11	10	1	97	23	21	25	11	14	0
20% Acetone	2	100	3	1600	123	58	40	23	55	34
Buffer 5.75 pH	11	12	6	160	50	12	77	3	50	33
10^−2^M H_2_SO_4_	29	110	110	179	108	35	11	49	40	1
10^−2 ^M HClO_4_	39	102	9	120	29	9	23	29	10	2
10^−1 ^M HCl	143	111	9	133	52	36	55	200	11	0
10^−1 ^M HNO_3_	34	127	70	142	110	38	16	84	0	11
10^−2 ^M HNO_3_	166	104	110	117	42	102	80	45	57	156
10%HCOOH	91	–	24	47	24	27	78	58	32	–

**Figure 6:**
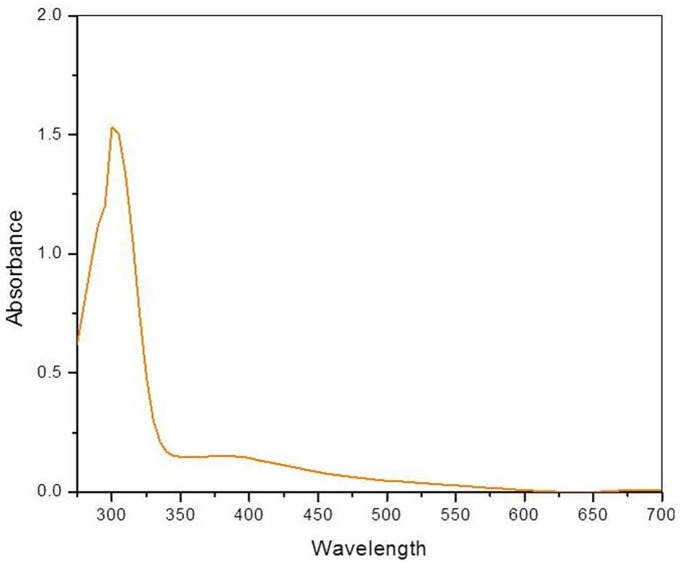
UV-visible spectra of POMA-MoP/Ag nanocomposite.

The optical property of the POMA-MoP/Ag was examined using UV–vis spectrophotometer at room-temperature and shown in **Fig. 6**. To measure the UV–vis absorption, the POMA-MoP/Ag were dispersed in distilled water and measured. The obtained UV absorption exhibits a well-defined exciton band at 300 nm, a characteristic and corresponding peak to single bulk compound and other peak related with impurities were not observed in the spectrum which confirms that the synthesized compound is in pure form.

### Preparation of Hg^2+^ Ion-selective Membrane

In this study, POMA-MoP/Ag cation-exchanger was also used for the preparation of heterogeneous ion-selective membrane electrode. Sensitivity and selectivity of the ion-selective membranes electrode depend upon the nature of electro-active material, membrane composition and physico-chemical properties of the membranes employed. A number of samples of the POMA-MoP/Ag nanocomposite membrane were prepared (Table 2) with different amount of composite material and PVC and checked for the mechanical stability, surface uniformity, materials distribution, cracks and thickness, etc. But the membranes obtained with 25% PVC (w/w) were found suitable.

**Table 2:** Characterization of ion-exchange membrane.

POMA-MoP/Agcomposite material	Thickness of themembrane (mm)	Water content as % weightof wet membrane	Porosity	Swelling of % weightof wet membrane
M-1	0.37	2.0000	2.7020×10^−4^	0.010
M-2	0.39	2.4896	1.5384×10^−4^	0.006
M-3	0.40	1.1152	7.5000×10^−5^	0.003

**Figure 7:**
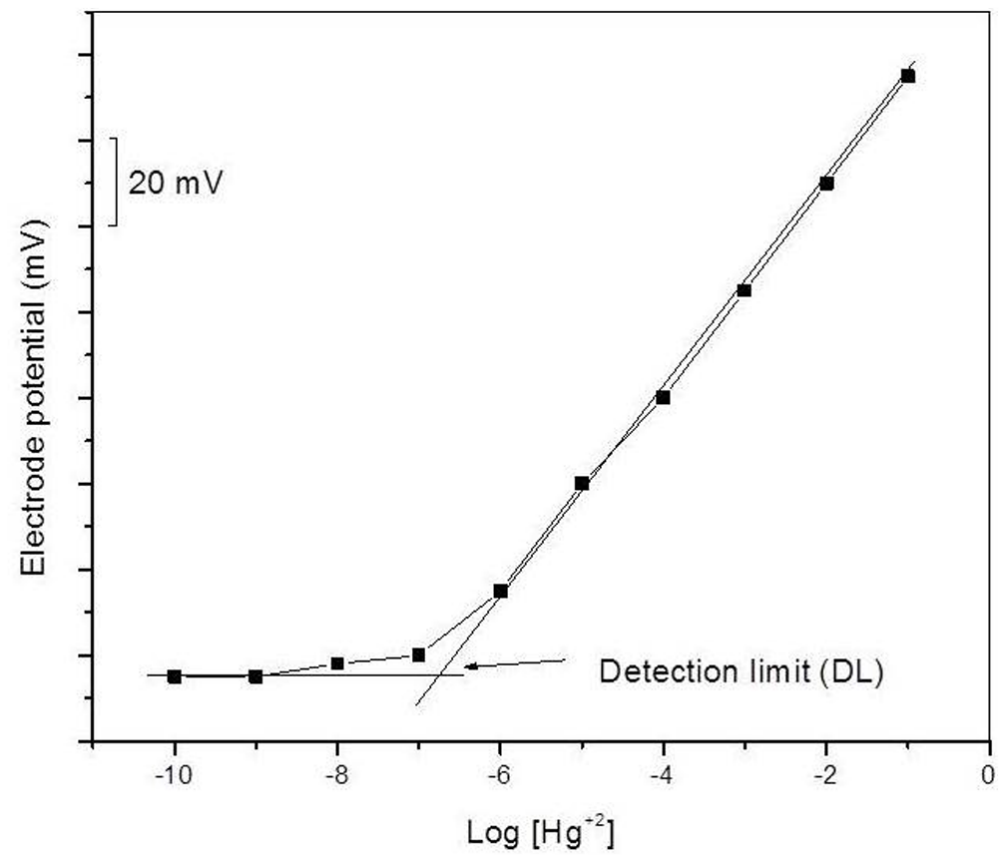
Calibration graph of Hg(II) ion selective POMA-MoP/Ag embedded membrane electrode.

The heterogeneous precipitate Hg(II) ion-selective membrane electrode obtained from POMA-MoP/Ag material gives linear response in the range 1 ×10^−1 ^M to 1×10^−6 ^M. Suitable concentration were chosen for sloping portion of the linear curve. The limit of detection determined from the intersection of the two extrapolated segments of the calibration graph [34] was found to be 8 ×10^−6 ^M, and thus the working concentration range is found to be 1 ×10^−1 ^M to 8 ×10^−6 ^M (**Fig. 7**.) for Hg^2+^ ions with a Nernstian slope of 28.5 mV per decade change in Hg^2+^ion concentration. The slope value is near to Nernstian value, 29.6 mV per concentration decade for divalent cation [35].

**Figure 8:**
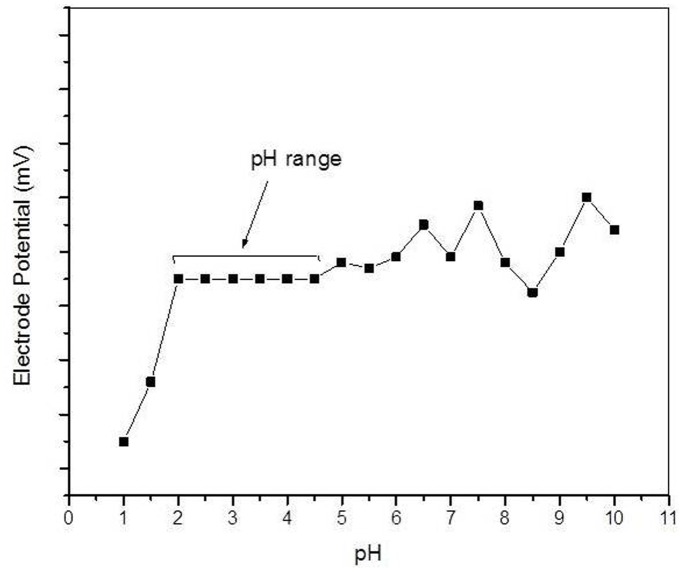
pH curve of POMA-MoP/Ag membrane ion selective electrode.

pH effect on the potential response of the membrane electrode were measured for a fixed (1×10^−2 ^M) concentration of Hg^2+^ ions in different pH values. It is clear that membrane potential remains unchanged with in the pH range 2.0–4.5 (**Fig. 8**), known as working pH for this membrane electrode in acidic medium.

**Figure 9:**
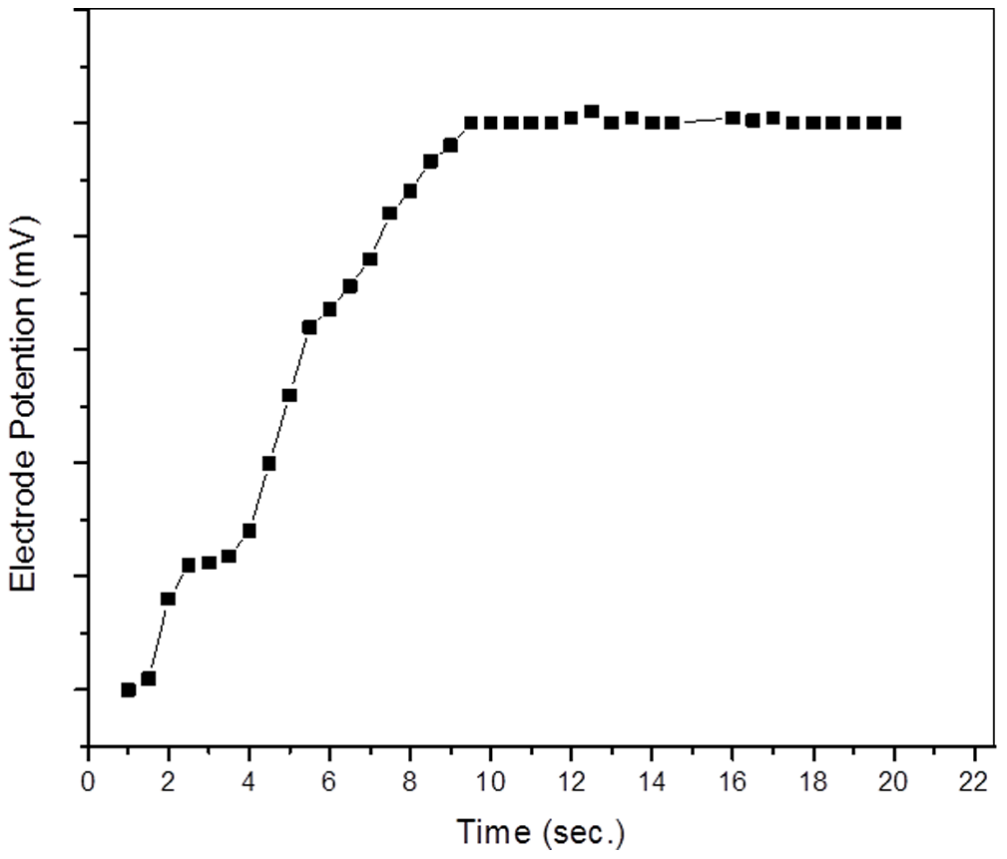
Response time curve for Hg(II) ion-selective membrane electrode based on POMA-MoP/Ag.

Another important factor is the promptness of the response of the ion-selective membrane electrode. The average response time is defined as the time required for the membrane to reach a stable potential. It is clear that the response time of the membrane sensor is found to be ∼10 s (**Fig. 9**).

The membrane electrode could be successfully used upto 3×1/2 months without any notable drift in potential during which the potential slope is reproducible within ±1 mV per concentration decade. Whenever a drift in the potential is observed, the membrane is re-equilibrated with 0.1 M metal nitrate solution for 4–5 days.

The selectivity coefficients, 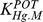 of various differing cations for the Hg(II) ion selective POMA-MoP/Ag nanocomposite membrane were determined by the mixed solution method [36]. The selectivity coefficient indicates the extent to which a foreign ion (M^n+^) interferes with the response of the membrane towards its primary ions (Hg^2+^). By examine the selectivity coefficient data given in **Table 3**, it is clear that the membrane is selective for Hg(II) in presence of interfering cations.

**Table 3:** The selectivity coefficients (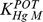) of various interfering cations (M^n+^).

Metal Ions	Selectivity coefficient values
Mg^2+^	0.001
Sr^2+^	0.004
Zn^2+^	0.005
Hg^2+^	0.008
Pb^2+^	0.005
Cu^2+^	0.001
Ca^2+^	0.003

The practical utility of the proposed membrane sensor assembly was tested by its use as an indicator electrode in the potentiometric titrations of Hg(II) with EDTA. The mercury selective membrane electrodes was employed as indicator electrodes in the titration of 1.0×10^−2^ M Hg(NO_3_)_2_ solutions against 1.0×10^−2^ M EDTA solution as a titrant. For this, 5 ml of Hg(NO_3_)_2_ solution was pipette out in a beaker. The volume of beaker was raised upto 20 mL by DMW. The solution was titrated with EDTA solution and electrode potential was measured after each addition of 0.5 ml of EDTA solution. The necessary adjustment of pH was made before adding the titrant. The addition of EDTA causes a decrease in potential as a result of the decrease in the free Hg(II) ions concentration due to its complexation with EDTA. The amount of Hg(II) ions in solution can be accurately determined from the resulting neat titration curve providing a sharp rise in the titration curve at the equivalence points. Some parameters of ion selective membrane electrode reported in literature compared with present studies given in **Table 4** [37]–[41].

**Table 4:** Comparative table of Hg(II) selective potentiometric membrane electrode.

S.No	Sensing Material	Detectionlimit (M)	Linear range	Slope	Responsetime (s)	Reference
1	1,2-bis-(*N’*-benzoylthioureido)cyclohexane	2.5×10–6	10^−5^ to 0.1	28.1±0.6	50–100	36
2	2-Amino-6-purinethiol	4.4×10–8	7.0×10^−8^ to 1.0×10^−1^	30.0	10	37
3	2-Mercaptobenzimidazole	6.0×10–7	10^−6^ to 10^−1^	28.5	20–100	38
4	Calixarene derivative	4.5×10–7	5×10^−6^ to 10^−2^	28.7	20	39
5	1-furan-2-yl-4-(4-nitrophenyl)-2-phenyl-5H-imidazole-3-oxide	6.30×10−6	1.0×10^−1^ to 1.0×10^−5^	29.35	20	40
6.	polythiophene/tin phosphate	1 ×10^−7^	1×10^−1^ to 1×10^−7^	29.29	23	17
7	Poly(o-anisidine)molybdophosphate	About 1 ×10^−7^	1×10^−1^–8×10^−6^	28.5	10	Current work

## Conclusions

In this study, a novel silver embedded nano composite cation-exchanger POMA-MoP/Ag, having good ion-exchange capacity and thermal stability had been prepared successfully. This composite material was also utilized as an electro active component for the preparation of ion-selective membrane for the determination of Hg(II) ions in aqueous solution. The membrane showed a working concentration range 1×10^−1^–8×10^−6^ M, response time 10s, pH range 2–4.5, and selectivity in presence of other metal ions. The practical utility was determined as potentiometric sensor for the titration of Hg(II) using EDTA as a titrant. This is a material of interests for removal of water pollution in heavy metal ion source of Hg. Conductivity studies will be continuing in the next part of study.

## Acknowledgments

This paper was funded by King Abdulaziz University, under grant No. (D- 004/431). The authors, therefore, acknowledge technical and financial support of KAU.

## References

[1] Whittingham MS (1978) Chemistry of intercalation compounds - metal guests in chalcogenide hosts. Prog Solid State Chem 12: 41–99.

[2] Khan A, Asiri AM, Rub MA, Azum N, Khan AAP, et al (2012) Review on composite cation exchanger as interdicipilinary materials in analytical chemistry Int. J. Electrochem Sci 7: 3854–3902.

[3] Maclachan M, Manners I, Ozin GA (2000) New (inter)faces: Polymers and inorganic materials. Adv Mater 12: 675–681.

[4] Gomez-Romero P (2001) Hybrid organic-inorganic materials - In search of synergic activity. Adv Mater 13: 163–174.

[5] Liu YJ, DeGroot DC, Schindler JL, Kannewurf CR, Kanatzidis MG (1991) Intercalation of poly(ethylene oxide) in V_2_O_5_ xerogel. Chem Mater 3: 992–994.

[6] Enzel P, Bein T (1989) Intrazeolite synthesis of polythiophene chains. J Chem Soc Chem Commun 18: 1326–1327.

[7] Kanatzidis MG, Tonge LM, Marks TJ, Marcy HO, Kannewurf CR (1987) Insitu intercalative polymerization of pyrrole in feocl - a new class of layered, conducting polymer inorganic hybrid materials. J Am Chem Soc 109: 3797–3799.

[8] Bissessur R, DeGroot DC, Schindler JL, Kannewurf CR, Kanatzidis MG (1993) Inclusion of poly(aniline) into MoO_3_ . J Chem Soc Chem Commun 8: 687–689.

[9] Disalvo FJ (1990) Solid-state chemistry - a rediscovered chemical frontier. Science 247: 649–655. 17771880 10.1126/science.247.4943.649

[10] Hutchison JC, Bissessur R, Shriver DF (1996) Conductivity anisotropy of polyphosphazene-montmorillonite composite electrolytes. Chem Mater 8: 1597–1599.

[11] Ibrahim MA, Lee BG, Park NG, Pugh JR, Eberl DD, et al (1999) Synthesis of new oligothiophene derivatives and their intercalation compounds: orientation effects. Synth Met 105: 35–42.

[12] Bissessur R, White W (2006) Novel alkyl substituted polyanilines/molybdenum disulfide nanocomposites. Mater Chem Phys 99: 214–219.

[13] Xu BH, Lin BZ, Sun DY, Ding C, Liu XZ, et al (2007) Preparation and characterization of organic-inorganic poly(ethylene glycol)/WS2 nanocomposite. Mater Res Bull 42: 1633–1639.

[14] Murray RW (1992) Molecular design of membrane surfaces. Wiley: New York.

[15] Finklea HO, Bard AJ, Rubinstein I (1996) Electroanalytical chemistry. Marcel Dekker: New York, p.19.

[16] Ling JLW, Khan A, Saad B, Ab Ghani S (2012) Electro polymerized 4-vinyl pyridine on 2B pencil graphite as ionophore for cadmium (II). Talanta 88: 477–483. 22265529 10.1016/j.talanta.2011.11.018

[17] Khan A, Asiri AM, Khan AAP, Rub MA, Azum N, et al (2013) Sol–gel synthesis and characterization of conducting polythiophene/tin phosphate nano tetrapod composite cation-exchanger and its application as Hg(II) selective membrane electrode. J Sol-Gel Sci Technol 65: 160–169.

[18] Zen JM, Senthil Kumar A, Tsai DM (2003) Recent updates of chemically modified electrodes in analytical chemistry. Electroanalysis 15: 1073–1084.

[19] Sadakane M, Steckhan E (1998) Electrochemical properties of polyoxometalates as electrocatalysts. Chem Rev 98: 219–237. 11851504 10.1021/cr960403a

[20] Muller A, Kogerler P, Kuhlmann C (1999) A variety of combinatorially linkable units as disposition: from a giant icosahedral Keplerate to multi-functional metal-oxide based network structures. Chem Commun 15: 1347–1358.

[21] Lira-Cantu M, Gomez-Romero P (1998) Electrochemical and chemical syntheses of the hybrid organic-inorganic electroactive material formed by phosphomolybdate and polyaniline. Application as cation-insertion electrodes. Chem Mater 10: 698–704.

[22] Kulkarni MV, Viswanath AK (2005) Spectroscopic, thermal and electrical properties of sulphonic acids doped poly(o-anisidine) and their application as humidity sensor. Sens. Actuators B 107: 791–797.

[23] Alberty G, Constantino U (1970) Crystalline insoluble acid salts of tetravalent metals: X. Fibrous thorium phosphate, a new inorganic ion-exchange material suitable for making inorganic sheets. J. Chromatogr. 50: 482–486.

[24] De AK, Chowdhury K (1974) The most widely used ionization techniques in liquid chromatography–mass spectrometry. J. Chromatogr. 101: 63–69.

[25] Vaillant J, Lira-Cantu M, Cuentas-Gallegos K, Casan-Pastor N, Gomez-Romero P (2006) Chemical synthesis of hybrid materials based on PAni and PEDOT with polyoxometalates for electrochemical supercapacitors. Prog Solid Stat Chem 34: 147–159.

[26] Khan AA, Khan A (2007) Inamuddin (2007) Preparation and characterization of a new organic-inorganic nano-composite poly-o-toluidine Th(IV) phosphate: Its analytical applications as cation-exchanger and in making ion-selective electrode. Talanta 72: 699–770. 19071675 10.1016/j.talanta.2006.11.044

[27] Srivastava SK, Jain AK, Agarwal S, Singh RP (1978) Studies with inorganic ion-exchange membranes. Talanta 25: 157–159. 18962228 10.1016/0039-9140(78)80105-2

[28] Amarchand S, Menon SK, Agarwal YK (1998) Water hardness determination using Mg(II) ion selective electrodev. Indian J Chem Technol 5: 99–103.

[29] Duval C (1963) Inorganic Thermogravimetric Analysis, Elsevier:Amsterdam, p.315.

[30] Rao CNR (1963) Chemical Applications of Infrared Spectroscopy, Academic Press: New York, p355.

[31] Rao CNR (1963) Chemical Applications of Infrared Spectroscopy, Academic Press: New York, p.338.

[32] Biju V, Sugathan N, Vrinda V, Salini SL (2008) Estimation of lattice strain in nanocrystalline silver from X-ray diffraction line broadening. J Mater Sci 43: 1175–1179.

[33] Amini MK, Mazloum M, Ensaf AA (1999) Lead selective membrane electrode using cryptand(222) neutral carrier, Fresenius. J Anal Chem 364: 690–693.

[34] Demirel A, Doğan A, Canel E, Memon S, Yilmaz M, et al (2004) Hydrogen ion-selective poly(vinyl chloride) membrane electrode based on a p-tert-butylcalix[4]arene-oxacrown-4. Talanta 1: 123–129. 10.1016/S0039-9140(03)00414-4 18969273

[35] IUPAC Analytical Chemistry Division (1994) Commission on Analytical Nomenclature. Pure Appl Chem 66: 2527.

[36] Umezawa Y, Bühlmann P, Umezawa K, Tohda K, Amemiya S (2000) Selectivity coefficients of ion-Selective electrodes part I. inorganic cations. Pure Appl. Chem. 72: 1851–2082.

[37] Jumal J, Yamin BM, Ahmad M, Heng LY (2012) Mercury ion- selective electrode with self-plasticizing poly(n-buthylacrylate) membrane based on 1,2-bis-(n-benzoylthioureido)cyclohexane as ionophore. APCBEE Procedia 3: 116–123.

[38] Gupta VK, Singh AK, Khayat MA, Gupta B (2007) Neutral carriers based polymeric membrane electrodes for selective determination of mercury (II). Anal Chim Acta 590: 81–90. 17416226 10.1016/j.aca.2007.03.014

[39] Mazloum M, Amini MK, Baltork IM (2002) Mercury selective membrane electrodes using 2-mercaptobenzimidazole, 2-mercaptobenzothiazole and hexathiacyclooctadecane carriers. Sens Actuators, B 63: 80–85.

[40] Lu J, Tong X, He X (2003) A mercury ion-selective electrode based on a calixarene derivative containing the thiazole azo group. J Electroanal Chem 540: 111–117.

[41] Mahajana RK, Puria RK, Marwahab A, Kaur I, Mahajan MP (2009) Highly selective potentiometric determination of mercury(II) ions using 1-furan-2-yl-4-(4-nitrophenyl)-2-phenyl- 5H-imidazole-3-oxide based membrane electrodes. J Hazard Mater 167: 237–243. 19185990 10.1016/j.jhazmat.2008.12.107

